# Determination of Polycyclic Aromatic Hydrocarbons (PAHs) and Phthalates in Human Placenta by Mixed Hexane/Ether Extraction and Gas Chromatography–Mass Spectrometry/Mass Spectrometry (GC-MS/MS)

**DOI:** 10.3390/metabo13090978

**Published:** 2023-08-29

**Authors:** Lin Tao, Yingkuan Tian, Dengqing Liao, Songlin An, Wei Chen, Xiang Liu, Pei Xu, Xubo Shen, Yuanzhong Zhou

**Affiliations:** Key Laboratory, School of Public Health, Zunyi Medical University, No.6 Xuefu West Road, Xinpu Street, Honghuagang District, Zunyi 563006, China

**Keywords:** pregnant women, placenta, pretreatment, triple quadrupole gas chromatography–mass spectrometry (GC-MS/MS), internal standard method

## Abstract

In this study, we evaluated the extraction effect of three different extractants, namely hexane + ether (*v*/*v* = 3:1), acetonitrile and ethyl acetate, on polycyclic aromatic hydrocarbons (PAHs) and phthalic acid esters (PAEs) in placenta detected and analysed by triple quadrupole gas chromatography–mass spectrometry (GC-MS/MS). The results showed that n-hexane + ether (*v*/*v* = 3:1) had the highest extraction efficiency. Under the optimal conditions, the limits of detection (LOD) for the 10 PAHs were 0.003–0.0167 μg/L with relative standard deviations (RSD) of 1.4–5.48% and detection rates of 68.19–107.05%, and the correlation coefficients were (R^2^, 0.9982–0.9999). The LODs for the nine PAEs were 0.0015–3.5714 μg/L and the correlation coefficients were (R^2^, 0.9982–0.9999). The limits of detection (S/N = 3) for the nine PAHs were 0.0015–0.5714 μg/L with relative standard deviations (RSD) of 3.15–8.37%, and the detection rates were 80.45–112.59% with correlations of (R^2^, 0.9972–0.9998). The method was applied to the analysis of PAHs and phthalates in placenta samples from pregnant women. The method’s accuracy and applicability were demonstrated. In comparison with other methods for the detection of PAEs and PAHs, the method proposed in this paper has a wider linear range, lower minimum detection limit and comparable recovery with good correlation. This paper is dedicated to providing another method for improving the performance of extracting solid tissues.

## 1. Introduction

In recent years, China’s rapid industrialisation and urbanisation have led to serious polycyclic aromatic hydrocarbon (PAH) and phthalate (PAE) pollution. PAHs and phthalates are persistent organic pollutants (POPs) that cause cancer and endocrine disruption [1]. Air pollution and fuel emissions are sources of exposure to PAHs. Cosmetics and plasticisers are sources of exposure to PAEs. PAHs and PAEs can enter the body through inhalation, ingestion and dermal contact and are excreted in urine and faeces. Studies have shown that PAH and PAE can cross the placenta from maternal serum into the foetal circulation [2,3], with potential implications for maternal and child health [4,5,6,7,8,9]. The placenta is highly sensitive to EEDs because it is rich in hormone receptors. Upon exposure of pregnant women to PAEs, their metabolites may cross the placenta and interfere in placental function through disruption of hormonal receptors. Placental dysfunction is known to result in foetal failure to thrive and even foetal death. Animal and in vitro studies have shown that possible mechanisms of abnormal placental function resulting from PAE exposure during pregnancy include infiltration/confluence [10,11,12], oxidative stress [13,14,15], cell differentiation/apoptosis [16,17,18], hormone secretion [19] and lipid accumulation [20]. Furthermore, a growing body of epidemiological and animal evidence shows that PAHs can cause placental structural and functional abnormalities through their effects on trophoblast invasion [21], migration [22], proliferation [23], apoptosis [24], and other functions that influence foetal growth and development. However, as information on placental exposure to PAH and PAE is lacking, evidence on whether these environmental pollutants affect placental structural function is incomplete. Currently, human PAH and PAE detection focuses on urine, blood, and hair [25,26,27,28,29], with few reports of detection in placenta and amniotic fluid. The triple-quadrupole gas chromatography–mass spectrometer (GC-MS-MS) is an analytical instrument used in the field of chemistry and is widely used for the detection of pesticide residues, especially for the detection of multiple residues and for the detection of organic contamination of the environment and organic contamination of food. It has high sensitivity, strong qualitative and quantitative capability, stable and durable performance, the ability to detect large batches, short detection time and simple operation for the analysis of detection data. In this work, we develop a protocol based on GC-MS-MS for the detection of PAHs and PAEs in bulk, as well as a robust method for the pretreatment of urine, which has been used with satisfactory results in several publications [30,31,32,33]. However, matrix effects affecting the detection of PAHs and PAEs in the placenta are more complex, with a greater variety of chemicals in placental tissues (e.g., clotting factors, hormones, enzymes and amino acids) and lower concentrations of PAHs and PAEs, as well as, more importantly, the fact that solid-state processing is more complex and difficult than liquid processing (solid–liquid conversion requires special treatments such as rapid freezing, grinding, etc.), and thus new pretreatment protocols need to be developed. Therefore, this paper proposes further investigating the placenta pretreatment method based on the group’s developed urine pretreatment protocol, aiming at the batch treatment of PAHs and PAEs.

## 2. Materials and Methods

### 2.1. Study Population and Specimens

The study population was drawn from the Zunyi Birth Cohort (ZBC), based on the National Key Research and Development Project “Environmental and behavioural factors on embryonic development and pregnancy based on internal and external exposure monitoring”. The study was approved by the Ethics Committee of Zunyi Medical University (No. [2019] H-005), and each participating pregnant woman voluntarily signed an informed consent form.

### 2.2. Laboratory Instruments and Consumables

Main instruments and consumables: fume cupboard, low-speed centrifuge, multi-tube vortex mixer, autosampler vials with brown thread, 250 μL cannulas, blue open screw caps, autosampler needles, chromatographic columns, pH meter, water bath, mass spectrometer (9000-7010D). Standard reagents: 1-Naphthol (1-OHNAP), 2-Naphthol (2-OHNAP), 2-Hydroxyfluorene (2-HFLU), 9-Hydroxyfluorene (9-HFLU), 9-Hydroxyphenanthrene (9-HPH), 1-Hydroxypyrene (1-OHPYR), 1-Hydroxyphenanthrene (1-OHPH), 2-Hydroxyphenanthrene (2-OHPH), 3-Hydroxyphenanthrene (3-OHPH), 4-Hydroxyphenanthrene (3-OHPH), 4-Hydroxyphenanthrene (3-OHPH), Monoethyl phthalate (mEP), monomethyl phthalate (MMP), monooctyl phthalate (MOP), monobenzyl phthalate (mBzP), monoisobutyl phthalate (MIBP), monobutyl phthalate (mBP), Mono(5-carboxy-2-ethylpentyl) phthalate (MECPP), Mono(2-ethyl-5-hydroxyhexyl) phthalate (MEHHP), Mono(2-ethyl-5-oxohexyl) phthalate (MEHHP), Mono(2-ethyl-5-oxohexyl) phthalate (MECPP) 1-Naphthol-2, 3,4,5,6,7,8-d7, 1-Hydroxypyrene-d9, Mono (2-ethyl-5-hydroxyhexyl) phthalate 13c (MEHPP), Mono (2-ethyl-5-hydroxyhexyl) phthalate 13c (MECPP), Monobutyl (mBP), monobutyl (5-carboxy-2-ethylpentyl) phthalate (MECPP), monobutyl (5-carboxy-2-ethylpentyl) phthalate (MEHPP), hexyl phthalate 13c (MEHP-C4) and mono (2-ethyl-5-hydroxyhexyl) phthalate 13c (MEHHP-C4). Other reagents: silylation reagent, magnesium sulphate, β-glucuronidase, sodium acetate, hexane, acetonitrile, ethyl acetate and ether. All reagents, experimental apparatus and loading conditions are detailed in previous publications of this research group [34,35].

### 2.3. Preparation of Storage Solutions

The concentrations for each compound are presented in Table S1. 2-OHPh and 3-OHPh were procured directly in 10 mL volumes with a concentration of 10 µg/mL. Sixteen brown 10 mL volumetric flasks labelled 2, 3, 4, 7, 8, 9, 10, 11, 12, 13, 14, 19, and 20 were utilized. To these pre-filled flasks, 0.5 mL of acetonitrile was added and then transferred to the corresponding sample flask already containing acetonitrile, as previously described. Using a pipette, transfer 0.5 mL of acetonitrile into the prefilled flask and then into the corresponding sample flask containing acetonitrile as previously described. Gently blow to mix. Take 6 brown 2 mL sample vials numbered 1, 5, 6, 16, 17, and 18. Pipette 200 µL of acetonitrile into each of the vials containing the samples and gently blow to mix well. Then, pipette 200 µL of acetonitrile into the corresponding sample vials mentioned earlier to make 1 mL. Then, 450 µL of acetonitrile was added to sample vials 16, 17, and 18 to obtain a volume of 1.25 mL. The solution should be stored away from light at a temperature of −20 °C.

### 2.4. Internal Standard Liquid

A total of 480 µL of 1-OHP-d9 reservoir solution at a concentration of 0.25 mg/mL was mixed with 240 µL of 1-OHNa-d7 reservoir solution at a concentration of 5 mg/mL, 1.2 mL of MEHP-c4 at a concentration of 100 μg/mL and 1.2 mL of MEHHP-c4 internal standard at a concentration of 100 μg/mL, respectively. Then, 2 mL of MEHHP-c4 internal standard was added at a concentration of 100 μg/mL, and the solution was fixed with acetonitrile to 24 mL to give a mixed internal standard solution of the four, with a final concentration of 50.9 μg/mL for 1-OHNa-d7 and 5 μg/mL for 1-OHP-d9, MEHP-c4 and MEHHP-c4, as shown in Table S2.

### 2.5. Mixed Standard Solution (Chemistry)

Mixing 7: take a 10 mL centrifuge tube, add 1 mL of acetonitrile labelled Mixing 7, use 20 µL and 50 µL pipettes to pipette the reservoir solution of different standards into the above mentioned 10 mL centrifuge tube and then make up to 5 mL with acetonitrile and mix on the vortexer, see Table S3. Mixing 6: take 1.5 mL of Mixing 7 and 1.5 mL of acetonitrile to the Mixing 6 tube and mix on a vortexer for 1 min, 3x. Mixing 4: take 2 mL of Mixing 5 and 2 mL of acetonitrile to the Mixing 5 tube and mix for 15 s, 3x. MixLabel 5: Add 1 mL of MixLabel 7 and 4 mL of acetonitrile to the MixLabel 5 tube, mix for 15 s on the vortexer 3 times. MixLabel 4: Add 2 mL of MixLabel 5 and 2 mL of acetonitrile to the MixLabel 4 tube, mix for 15 s on the vortexer 3 times. MixLabel 3: Take 1 mL of MixLabel 4 and 4 mL of acetonitrile and add to the MixLabel 3 tube, mix for 15 s on the vortexer 3 times. MixLabel 2: Take 2 mL of MixLabel 3 and 2 mL of acetonitrile and add to the MixLabel 2 tube, mix for 1 min. MixLabel 2: Take 2 mL of MixLabel 3 and 2 mL of acetonitrile and add to the Mixing Standard 2 tube and mix for 15 s on the vortexer 3 times. Mixing Standard 1: 1.5 mL Mix Standard 2 and 1.5 mL acetonitrile are added to the Mix Standard 1 tube and mixed on the vortexer for 15 s, 3 times. Control 0: 3 mL acetonitrile is added to the Control 8 tube as a blank control. See Table S4.

### 2.6. Standard Series

Take eight 10 mL round-bottomed plastic centrifuge tubes labelled C0, B1~B7. Add 1462.5 µL of pure water, 37.5 µL of the above mixing standards (Mixing Standard 1 for B1, Mixing Standard 2 for B2, Mixing Standard 3 for B3, Mixing Standard 4 for B4, Mixing Standard 5 for B5, Mixing Standard 6 for B6, Mixing Standard 7 for B7) to each of the tubes from B1 to B7 and Mixing Standard 7 for B0, Mixing Standard 0 and Mixing Standard 0 and Mixing Standard Series in B0, and then mix to the standard series concentration. B0 tube with 1462.5 µL of pure water, 37.5 µL of the above control 0 and mix well to formulate the standard series concentration. See Table S5.

### 2.7. Placenta Sample Pre-Processing Protocol

Scheme 1 (extractant: acetonitrile): Step 1: Take the placenta sample out of the −80 °C fridge and place it in the 4 °C fridge overnight to thaw. Step 2: Dry the placental tissue in an oven at 80 °C and grind to a powder with liquid nitrogen. Step 3: A 50 mg sample of ground tissue is accurately weighed into a 5 mL centrifuge tube and numbered. Step 4: Add 1 mL of acetonitrile to the sample tube and shake well to mix. Step 5: Add 10 µL of internal standard solution and 10 µL of β- glucuronidase/sulphatase enzyme to both sample and standard tubes and leave in a water bath at 37 °C overnight (12–16 h). Step 6: Accurately weigh a new batch of 5 mL centrifuge tubes using an analytical balance (weight 1). Step 7: Add 1 mL of MgSO_4_-7H_2_O one by one and shake on a multi-tube vortex mixer for 10 min at 2500 r/min until no precipitate is present. Step 8: Add 1 mL of acetonitrile one by one, centrifuge for 10 min (4000 rpm) and remove the supernatant to a new 5 mL centrifuge tube (approximately 0.3 mL, the supernatant cannot be removed). Add 1 mL of acetonitrile to each tube again, mix for 15 s in a multi-tube vortexer, centrifuge for 10 min (same speed as above) and remove the supernatant (approximately 1 mL). Step 9: Blow dry the total organic phase from the three extractions with high purity nitrogen and weigh (weight 2), fat weight = weight 2-weight 1. Step 10: Add 300 µL of acetonitrile to the weighed centrifuge tube and cover with a cap. Step 11: Add 100 µL of silylation reagent to the inner cannula and place in a water bath at 90 °C for 45–60 min to allow full derivatisation. Cool at room temperature for 30 min and then assay on the machine. Scheme 2 (extractant: ethyl acetate): replace all the extractants in scheme 1 with ethyl acetate and leave the rest of the operation unchanged. scheme 3 (extractant: hexane + ether; volume ratio = 4:1): replace all the extractants in scheme 1 with hexane + ether, otherwise the operation remains unchanged. scheme 4 (extractants: n-hexane + ether; *v*/*v* = 4:1 and ethyl acetate): a. replace the extractant with hexane + ether for any two extractions in scheme 1 and ethyl acetate for the remaining one, otherwise the operation remains unchanged; b. replace any two extractants in scheme 1 with ethyl acetate and the remaining one with n-hexane + ether, otherwise the operation remains unchanged.

### 2.8. Chromatographic Conditions

The temperature of the inlet was 250 °C, and the shunt mode was not used. The carrier gas was 99.999% helium, and the flow rate was kept at 1.2 mL/min. The sample volume of each autosampling was 1 μL. The temperature was maintained at 250 °C for 3 min, then gradually raised at the same rate to 210 °C for 5 min, and finally to 280 °C for another 5 min. This entire process took 33 min. The ion source for mass spectrometry was the electron bombardment (EI) source. The injection temperature was 250 °C, while the ion source temperature was 280 °C, and the gain was set to 1. Individual standards served as samples for characterisation, and the retention time of each compound was determined based on the product ion pair of each compound. For further details, refer to Table S6 for characterisation data.

### 2.9. Sampling Conditions

Firstly, check for any sediment in the vial’s cannula. If there is no sediment, then the sample is suitable for injection. If there is sediment, then the sample must not be injected to avoid clogging of the injection needle. Next, open the relevant software and edit the sequence. Finally, execute and save the data.

### 2.10. Analysis of Results

Quantitative analysis of each substance was carried out using the relevant software. Values that fell below the limit of detection (LOD) were adjusted according to the formula LOD/√2 to eliminate the effect of placental dilution. To correct for possible variations in urinary creatinine levels, the concentration of PAH metabolite was adjusted using the concentration of urinary creatinine, resulting in a metabolite creatinine-corrected concentration (μg/g Cr) of concentration of PAH or PAE metabolite (μg/L) divided by [concentration of urinary creatinine (mmol/L) × 113.12 (g/mol) × 10].

### 2.11. Principle and Quality Control

This approach predominantly employs the principle of solvent similarity to isolate the desired components. It encompasses a qualitative and quantitative assessment of the target compounds and ultimately relies on a comparative analysis to pinpoint the optimal extraction protocol. Our extraction methodology employs rigorous quality control measures. Initially, we identified the limit of detection (LOD) and limit of quantification (LOQ) for the target compounds. Three times the signal-to-noise ratio was established as the limit of detection (LOD) and ten times the signal-to-noise ratio as the limit of quantification (LOQ) of the method. Furthermore, the experimental procedure underwent quality control using a standard addition method, which consisted of adding low, medium, and high concentrations of the target compounds to 1.5 mL of urine sample. Six parallel samples were collected for each concentration, with an additional six parallel samples. Each batch of 6 samples with the same concentration was tested simultaneously with an additional 6 samples without the standard additive. This methodology was implemented to ascertain the spiked recovery (R) and relative standard deviation (RSD). The %RSD was subsequently utilised to evaluate the precision of the method.

### 2.12. Precautions

a. The numbering of the tubes should correspond to the numbering of the subjects, and should be written down in the lab notebook and then made into an Excel spreadsheet for easy reference and checking in the future. b. A total of 20 µL of internal standard solution and 20 µL of β-glucuronidase/sulphatase enzyme should be added with a white tip, and the tip of the tip should not be extended too deeply into the liquid surface when aspirating to prevent the liquid from being attached to the outer wall of the tip. c. Add MgSO_4_-7H_2_O and shake well until there is no precipitation. If there is precipitation when adding n-hexane + ether, then it needs to be mixed again on the vortexer. d. The supernatant cannot be taken completely, because there is a transparent layer above the coloured part; if this is taken and sucked out, it will lead to precipitation in the injection bottle, affecting the accuracy of the results, and may block the injection needle.

## 3. Results

### 3.1. Comparison of Different Extractants

If acetonitrile was used as extractant, the fit of the standard curve was around 0.75, and PAH were able to detect seven to eight metabolites, whereas phthalates could only detect two or three metabolites; if ethyl acetate was used as extractant, the fit of the standard curve was around 0.90, and PAH were able to detect eight or nine metabolites, whereas phthalates could only detect six or seven metabolites; if n-hexane + ether (volume ratio: 4:1) was used, the standard curve was around 0.90, and PAHs could detect eight or nine metabolites, while phthalates could detect only six or seven metabolites; when n-hexane + ether (volume ratio: 4:1) was used as extractant, the standard curve fit was above 0.99 and PAHs were able to detect 10 metabolites, while phthalates could only detect 9–10 metabolites; when a mixture of n-hexane + ether and ethyl acetate was used for extraction, the standard curve fit was around 0.90, and PAHs were able to detect eight or nine metabolites, while phthalates could only detect seven or eight metabolites. In conclusion, n-hexane + ether was the best extractant for PAHs and phthalates. See Table 1.

### 3.2. Sample Chromatogram

**Figure 1:**
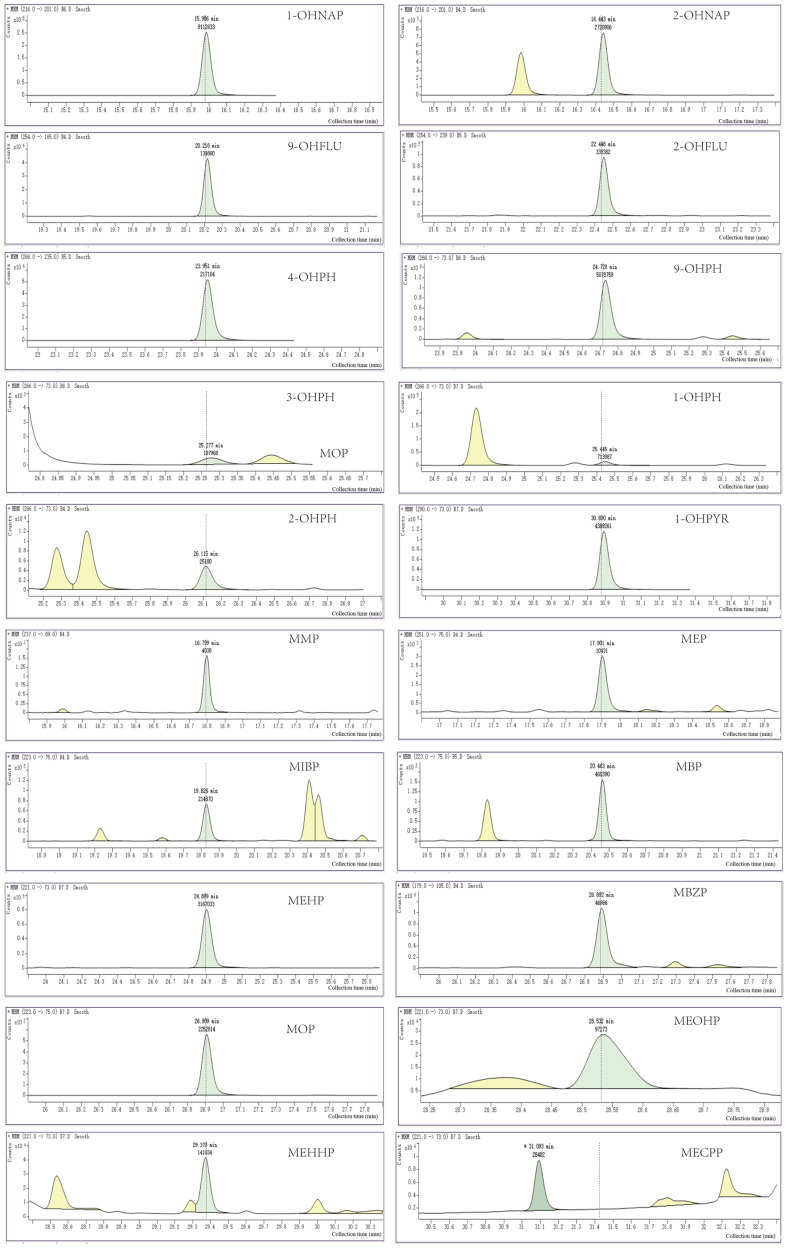
Chromatogram of PAHs and PAES in placenta.

The chromatograms were not plotted and the spiked recoveries were not counted when a mixture of acetonitrile, ethyl acetate, n-hexane + ethyl ether and ethyl acetate was used as extractant due to poor results. When using n-hexane + ether as extractant, a total of about 20 substances were detected, of which 10 were polycyclic aromatic hydrocarbon metabolites, namely 1-OHNAP, 2-OHFLU, 3-OHPHE, 9-OHPHE, 1-OHPHE, 2-OHNAP, 4-OHPHE, 1-OHPYR, 2-OHPHE, 9-OHFLU; 10 metabolites were identified: MECPP, MEOHP, MMP, MBP, MEHHP, MEP, MOP, MBZP, MEHP, and MIBP. Selection and analysis of the chromatograms revealed that the peaks of the 19 metabolites were more distinct, with the exception of MECPP, which was not evident. See Figure 1.

### 3.3. Spiked Recovery Rate

The spiked recoveries (R) for the 20 compounds ranged from 80% to 140%. Regression coefficients R^2^ > 0.997 were obtained for all of the pollutants except MECPP, and the experimental results showed that the limits of detection for most of the compounds were around 0.0001 mg/m^3^. RSD was 1–5, see Table 2.

## 4. Discussion

In this work, hexane and ether were finally selected as the main extractants, mainly because of the following: firstly, the chemical properties of hexane and ether are stable, and both can be dissolved in most organic solvents and have high volatility; secondly, hexane is a very efficient extractant without any side products. Conversely, both acetonitrile and ethyl acetate have by-products in the extraction process. For example, acetonitrile undergoes a typical nitrile reaction in the extraction process, and ethyl acetate is also prone to phosphoric acid hydrolysis reaction. Studies have shown that nitrile and phosphate hydrolysis inhibit extraction. Studies suggest that spiking a sample with a standard and determining recovery is the most common laboratory quality control method [36]. Previously established compliance limits for spiked recoveries are relatively strict, and generally require 95% to 105%. However, due to the complexity of the environmental sample matrix and the large concentration differences, the determination is prone to errors which can have an impact on the accuracy of the results. In recent years, quality control indicators have been developed to relax the spiking recovery requirements from a practical perspective: for example, some researchers have proposed spiking recovery control limits of 70% [34], 70–130% [35] and 60–140% [36]. Using this method, 10 PAHs and 9–10 PAEs were detected with recoveries ranging from 80% to 140%. The method has a wider range of spiked recoveries. In addition, the limit of detection of a compound is another criterion for evaluating an assay programme. The lowest detection limit in this method was 0.0015 μg/L for PAEs and 0.0003 μg/L for PAHs. Most studies have reported minimum limits of detection (LODs) to be greater than 0.005 g/L for PAEs [37,38,39] and 0.2 μg/L for PAHs [40,41,42], and the linear range of the proposed method is wider than that of other methods for the detection of PAEs and PAHs. The advantages of this work are (1) the detection of human placental tissue, (2) sophisticated detection instrumentation and inexpensive extraction reagents, and (3) combined extraction and detection of PAHs and PAEs in large quantities. However, in this pretreatment programme, the level of nitrogen injection must be strictly controlled, and attention should be paid to controlling the nitrogen rate during nitrogen injection (generally at a slow, uniform rate), as well as to aspirating the supernatant during extraction, which should not be pumped to the end, but should be kept at a height of 2 mm; otherwise, crystals will easily precipitate.

## 5. Detection Method Evaluation

Qualitative analysis: The sample underwent gas chromatography/mass spectrometry (GC/MS). Mass spectrometry enables the identification of target compounds if the retention time of the detected peaks coincides with that of the standard, and the selected ions are present in the mass spectra of the samples after subtracting the background, while the abundance ratio of the selected ions is consistent with that of the standard. Quantitative analysis: In this section, quantitative analysis was performed on the ions selected from Table 2 using the internal standard working curve method. The response value of the measured samples was obtained by deducting the response value of the blank experiment under the optimised conditions of gas chromatography and mass spectrometry. The concentration of each target compound in the samples was determined by plotting the standard working curve. The concentration of the components being measured should fall within the linear range for the experiment. Prior to conducting the experiment, it was necessary to perform a blank test to eliminate any possible interference from instruments or solvents.

## 6. Conclusions

Based on the pretreatment of urine proposed in this work, different extraction protocols were established, and the extraction efficiency of different extraction protocols was evaluated on the basis of standard curves and recoveries, and finally a relatively robust and efficient pretreatment protocol for placental tissue batches was developed, providing an important reference for the establishment of pretreatment protocols for the detection of organic contamination levels in human tissues.

## Figures and Tables

**Table 1:** Comparison of the extraction effect of different extractants.

Extractants	PAHS	PAEs	Standard Curve (R^2^ > 0.98)
Acetonitrile	1-OHNAP, 2-OHFLU, 3-OHPHE, 9-OHPHE, 1-OHPHE, 2-OHNAP, 4-OHPHE, 1-OHPYR	MBZP, MEHP, MIBP	0.75
Ethyl acetate	1-OHNAP, 2-OHFLU, 3-OHPHE, 9-OHPHE, 1-OHPHE, 2-OHNAP, 4-OHPHE, 1-OHPYR, 9-OHFLU	MBP, MEHHP, MEP, MOP, MBZP, MEHP, MIBP	0.90
n-hexane + ether	1-OHNAP, 2-OHFLU, 3-OHPHE, 9-OHPHE, 1-OHPHE, 2-OHNAP, 4-OHPHE, 1-OHPYR, 2-OHPHE, 9-OHFLU	MEOHP, MMP, MBP, MEHHP, MEP, MOP, MBZP, MEHP, MIBP	0.99
n-hexane + ether (1 extraction/2 extractions) and ethyl acetate (2 extractions/ extractions)	1-OHNAP, 2-OHFLU, 3-OHPHE, 9-OHPHE, 1-OHPHE, 2-OHNAP, 4-OHPHE, 1-OHPYR, 9-OHFLU	MMP, MBP, MEHHP, MEP, MOP, MBZP, MEHP, MIBP	0.90

**Table 2:** Detection limits, quantification limits, recoveries, and precision of 20 metabolites.

Compounds	Inner Label	Regression Equation	R^2^	LOD (μg/L)	LOQ (μg/L)	Recycling Rate (%)	RSD
1-OHNAP	1-OHNAP-D7	Y = 0.0367x + 0.0126	0.9998	0.0050	0.0167	107.05	1.40
2-OHNAP	1-OHNAP-D7	Y = 0.0509x + 0.0035	0.9999	0.0027	0.0090	105.71	2.27
9-OHFLE	1-OHNAP-D7	Y = 0.0371x − 0.0011	0.9998	0.0023	0.0078	94.15	2.85
2-OHFLE	1-OHNAP-D7	Y = 0.0663x − 0.0013	0.9998	0.0029	0.0098	91.99	3.18
4-OHPHE	1-OHNAP-D7	Y = 0.0409x + 6.1544	0.9999	0.0167	0.0556	90.08	5.01
9-OHPHE	1-OHNAP-D7	Y = 0.2914x − 0.0055	0.9982	0.0044	0.0147	105.01	4.99
1-OHPHE	1-OHNAP-D7	Y = 0.0383x − 0.0016	0.9998	0.0071	0.0236	81.09	4.96
3-OHPHE	1-OHNAP-D7	Y = 0.0188x − 0.0010	0.9999	0.0095	0.0316	84.50	5.48
2-OHPHE	1-OHNAP-D7	Y = 0.0171x − 4.7902	0.9999	0.0115	0.0385	68.19	2.41
1-OHPYR	1-OHPYR-D9	Y = 1.770x + 0.0213	0.9999	0.0003	0.0011	100.30	1.97
MMP	MEHP-C4	Y = 0.0037x + 5.9620	0.9986	0.0375	0.1250	80.45	8.37
MEP	MEHP-C4	Y = 0.9972x + 0.0202	0.9972	0.0288	0.0962	102.31	6.15
MIBP	MEHP-C4	Y = 0.1002x + 0.0813	0.9988	0.0023	0.0075	99.71	4.95
MBP	MEHP-C4	Y = 0.1681x + 0.1936	0.9982	0.0015	0.0051	92.64	4.77
MOP	MEHP-C4	Y = 0.1300x + 0.0021	0.9997	0.0625	0.2083	100.00	3.15
MEHP	MEHP-C4	Y = 0.1090x + 0.0234	0.9999	0.0326	0.1087	112.59	3.59
MEOHP	MEHHP-C4	Y = 0.0834x − 0.0876	0.9986	1.0714	3.5714	105.16	4.14
MBZP	MEHP-C4	Y = 0.0877x + 0.0351	0.9976	0.0405	0.1351	92.01	5.83
MEHHP	MEHHP-C4	Y = 0.2029x − 0.0621	0.9998	0.0103	0.1087	104.21	3.67

## Data Availability

Data associated with the present study can be accessed on request to the corresponding author. Data is not publicly available due to privacy.

## Supplementary Materials

The following supporting information can be downloaded at: https://www.mdpi.com/article/10.3390/metabo13090978/s1, Table S1: Preparation of PAHs and PAEs reservoirs, Table S2: Preparation of internal standard solution, Table S3: Preparation of Mixed Label 7, Table S4: Preparation of the remaining mixed standard solutions, Table S5: Standard Series Concentrations, Table S6: Information sheet for on-board checking of metabolites and internal markers.
