# Supplementary material for: Determination of Polycyclic Aromatic Hydrocarbons (PAHs) and Phthalates in Human Placenta by Mixed Hexane/Ether Extraction and Gas Chromatography–Mass Spectrometry/Mass Spectrometry (GC-MS/MS)
Source: Metabolites. 2023 Aug 29;13(9):978. doi: 10.3390/metabo13090978 (PMC10536546; doi:10.3390/metabo13090978)
Supplement: Supplementary file 1 [file metabolites-13-00978-s001.zip › metabolites-2578285-supplementary.pdf]

## Preparation of stock solutions

2-OHPh, 3-OHPh were obtained by direct purchase, packaged in 10 ml with a final concentration of 10 ug/ml. The specifications of the standards (pure) we purchased were the masses given in Table 4. Firstly, sixteen 10 ml brown volumetric flasks numbered 2, 3, 4, 7, 8, 9, 10, 11, 12, 13, 14, 19, 20. 0.5 ml of acetonitrile was pipetted into the packaged flasks using a 1 ml pipette gun, blown and mixed, and then pipetted into the corresponding sample flasks as above, and this was repeated four times, and then the flasks were fixed in acetonitrile to 10 ml. Six further 2 ml brown injection flasks, numbered 1, 5, 6. 16, 17, 18. Pipette 200 μL of acetonitrile into the packing bottle using a 200 μL pipette gun, blow and mix, then pipette into the corresponding sample vials mentioned above, repeat 4 times, then add 200 μL of acetonitrile to the sample vials to be diluted to 1 ml (Nos. 16, 17, 18 add 450 μL to be diluted to 1.25 ml). Store at -20°C. Protect from light. The final concentration of each compound is given in Table S1.

**Table S1.** Preparation of PAHs and PAEs reservoirs

| number | chemical | quantity (mg) | Acetonitrile volume (ml) | terminal concentration (mg/ml) |
|---|---|---|---|---|
| 1 | 1-OHNa | 10 | 1 | 10 |
| 2 | 2-OHNa | 100 | 10 | 10 |
| 3 | 2-OHFlu | 5 | 10 | 0.5 |
| 4 | 9-OHFlu | 10 | 10 | 1 |
| 5 | 9-OHPh | 10 | 1 | 10 |
| 6 | 1-OHPYR | 10 | 1 | 10 |
| 7 | 1-OHPH | 10 | 10 | 1 |
| 8 | 2-OHPH | - | - | 10 μg/ml |
| 9 | 3-OHPH | - | - | 10 μg/ml |
| 10 | 4-OHPh | 5 | 10 | 0.5 |
| 11 | MEHP | 100 | 10 | 10 |
| 12 | MEP | 100 | 10 | 10 |
| 13 | MMP | 100 | 10 | 10 |
| 14 | MOP | 100 | 10 | 10 |
| 15 | MBZP | 100 | 10 | 10 |
| 16 | MIBP | 100 | 10 | 10 |
| 17 | MBP | 100 | 10 | 10 |
| 18 | MECPP | 2.5 | 1.25 | 2 |
| 19 | MEHHP | 2.5 | 1.25 | 2 |
| 20 | MEOHP | 2.5 | 1.25 | 2 |
| 21 | 1-OHNa-d7 | 50.9 | 10 | 25 |

| | | | | |
|---|---|---|---|---|
| 22 | 1-OHP-d9 | 2.5 | 10 | 0.25 |
| 23 | MEHP-c4(1.2ml) | - | - | 100 μg/ml |
| 24 | MEHHP-c4(1.2ml) | - | - | 100 μg/ml |

**Preparation of the internal standard**

480 ul of 1-OHP-d9 stock solution at a concentration of 0.25 mg/ml was mixed with 240 ul of 1-OHNa-d7 stock solution at a concentration of 5 mg/ml, 1.2 ml of MEHP-c4 at a concentration of 100 ug/ml and 1.2 ml of MEHHP-c4 internal standard at a concentration of 100 ug/ml. 2 ml of MEHHP-c4 internal standard at a concentration of 100 ug/ml and the solution was made up to 24 ml with acetonitrile to give a The mixed internal standard solution of the four, 1-OHNa-d7 at a final concentration of 50.9 ug/ml and 1-OHP-d9, MEHP-c4 and MEHHP-c4 at a final concentration of 5 ug/ml, is shown in Table S2.

**Table S2.** Preparation of internal standard solution

| internal standard | Stock solution concentration （mg/ml） | stock solution （ml） | capacitation （ml） | Final concentration （μg/ml） |
|---|---|---|---|---|
| 1-OHNa-d7 | 25 | 0.02 | 10 | 50.9 |
| 1-OHP-d9 | 0.25 | 0.2 | | 5 |
| MEHP-c4 | 0.100 | 0.5 | | 5 |
| MEHHP-c4 | 0.100 | 0.5 | | 5 |

**Preparation of the Mixed Standard**

Preparation of Mixed Standard 7

Preparation of Mixed Standard 7: Take a 10 ml centrifuge tube, add 1 ml of acetonitrile labelled Mixed Standard 7, use 20 μl and 50 μl pipettes to transfer the reservoir solution of the different standards (for volumes see Table 5) into the above 10 ml centrifuge tube and then mix with acetonitrile to 5 ml on the vortexer to form Mixed Standard 7. See Table S3.

**Table S3.** Preparation of Mixed Label 7

| number | chemical | Stock solution concentration (mg/ml) | stock solution (μl) | Mixed scale 7 concentration (μg/ml) |
|---|---|---|---|---|
| 1 | 1-OHNa | 10 | 10 | 20 |
| 2 | 2-OHNa | 10 | 10 | 20 |
| 3 | 2-OHFlu | 0.5 | 20 | 2 |
| 4 | 9-OHFlu | 1 | 10 | 2 |

| | | | | |
|---|---|---|---|---|
| 5 | 9-OHPh | 1 | 10 | 2 |
| 6 | 1-OHPYR | 1 | 10 | 2 |
| 7 | 1-OHPh | 1 | 10 | 2 |
| 8 | 2-OHPh | 0.01 | 1000 | 2 |
| 9 | 3-OHPh | 0.01 | 1000 | 2 |
| 10 | 4-OHPh | 0.5 | 20 | 2 |
| 11 | MEHP | 10 | 10 | 20 |
| 12 | MEP | 10 | 10 | 20 |
| 13 | MMP | 10 | 10 | 20 |
| 14 | MOP | 10 | 10 | 20 |
| 15 | MBZP | 10 | 10 | 20 |
| 16 | MIBP | 10 | 10 | 20 |
| 17 | MBP | 10 | 10 | 20 |
| 18 | MECPP | 2 | 50 | 20 |
| 19 | MEHHP | 2 | 50 | 20 |
| 20 | MEOHP | 2 | 50 | 20 |

**Preparation of Mixed Label 2-Mixed Label 7 and Control 0**

Prepare seven 5 ml centrifuge tubes labelled: Mixed Label 2, Mixed Label 3, Mixed Label 4, Mixed Label 5, Mixed Label 6, Mixed Label 7 and Control 0. ② Mixed Label 6: Take 1.5 ml of Mixed Label 7 and 1.5 ml of acetonitrile and add to the Mixed Label 6 tube, vortex for 1 minute and then prepare Mixed Label 6. Mixing scale 5: Take 1 ml of mixing scale 7 and 4 ml of acetonitrile into the tube of mixing scale 5, vortex for 15 s and mix three times. (3) Mixing Standard 5: Take 2 ml of Mixing Standard 5 and 2 ml of acetonitrile into the tube of Mixing Standard 4, vortex for 15 s, mix 3 times and mix to make Mixing Standard 4. (5) Mixing Standard 3: Take 1 ml of Mixing Standard 4 and 4 ml of acetonitrile into the tube of Mixing Standard 3, vortex for 15 s, mix 3 times and mix to make Mixing Standard 3. (6) Mixing Standard 2: Take 2 ml of Mixing Standard 3 and 2 ml of acetonitrile and add to the tube of Mixing Standard 2. Vortex for 15s, 3x and mix to make Mixing Standard 3. Mix 15s, 3 times, formulated as Mixed Standard 2. (7) Mixed Standard 1: Take 1.5 ml of Mixed Standard 2 and 1.5 ml of acetonitrile added to the Mixed Standard 1 tube, mix on the vortexer 15s, 3 times, formulated as Mixed Standard 1. (8) Control 0: Take 3 ml of acetonitrile added to the Control 8 tubes, as blank control. See table S4.

**Table S4.** Preparation of the remaining mixed standard solutions

| standard solution | Add the mixing solution | Volume of acetonitrile（ml） |
|---|---|---|

| | | |
|---|---|---|
| Mixed Label 7 | Mixed Label 7 | |
| Mixed Label 6 | 1.5 ml Mixed Label 7 | 1.5 |
| Mixed Label 5 | 1 ml Mixed Label 6 | 4 |
| Mixed Label 4 | 2 ml Mixed Label 5 | 2 |
| Mixed Label 3 | 1 ml Mixed Label 4 | 4 |
| Mixed Label 2 | 2 ml Mixed Label 3 | 2 |
| Mixed Label 1 | 1.5 ml Mixed Label 2 | 1.5 |
| Control 0 | 0 | 3 |

**Standard Series Preparation**

Take eight 10ml round bottom plastic centrifuge tubes labelled C0, B1~B7. Add 1462.5μl of pure water, 37.5μl of the above Mixing Standards (Mixing Standard 1 for B1, Mixing Standard 2 for B2, Mixing Standard 3 for B3, Mixing Standard 4 for B4, Mixing Standard 5 for B5, Mixing Standard 6 for B6, Mixing Standard 7 for B7) to each of the tubes from B1 to B7, and Mixing Standard 1, Mixing Standard 2 and 37.5μl of the above Control 0 to B0, then mix well to form the standard series concentration. B0 tube with 1462.5 μl of pure water, 37.5 μl of the above Control 0 and mix well to formulate the standard series concentration. See table S5.

**Table S5.** Standard Series Concentrations

| chemicals | Series Concentration (μg/l) | | | | | | | |
|---|---|---|---|---|---|---|---|---|
| | C0 | B1 | B2 | B3 | B4 | B5 | B6 | B7 |
| 1-OHNa | 0 | 2.5 | 5 | 10 | 50 | 100 | 250 | 500 |
| 2-OHNa | 0 | 2.5 | 5 | 10 | 50 | 100 | 250 | 500 |
| 2-OHFlu | 0 | 0.25 | 0.5 | 1 | 5 | 10 | 25 | 50 |
| 9-OHFlu | 0 | 0.25 | 0.5 | 1 | 5 | 10 | 25 | 50 |
| 1-OHPh | 0 | 0.25 | 0.5 | 1 | 5 | 10 | 25 | 50 |
| 2-OHPh | 0 | 0.25 | 0.5 | 1 | 5 | 10 | 25 | 50 |
| 3-OHPh | 0 | 0.25 | 0.5 | 1 | 5 | 10 | 25 | 50 |
| 4-OHPh | 0 | 0.25 | 0.5 | 1 | 5 | 10 | 25 | 50 |
| 9-OHPh | 0 | 0.25 | 0.5 | 1 | 5 | 10 | 25 | 50 |
| 1-OHP | 0 | 0.25 | 0.5 | 1 | 5 | 10 | 25 | 50 |
| MEHP | 0 | 2.5 | 5 | 10 | 50 | 100 | 250 | 500 |
| MEP | 0 | 2.5 | 5 | 10 | 50 | 100 | 250 | 500 |
| MMP | 0 | 2.5 | 5 | 10 | 50 | 100 | 250 | 500 |
| MOP | 0 | 2.5 | 5 | 10 | 50 | 100 | 250 | 500 |
| MBZP | 0 | 2.5 | 5 | 10 | 50 | 100 | 250 | 500 |
| MIBP | 0 | 2.5 | 5 | 10 | 50 | 100 | 250 | 500 |
| MBP | 0 | 2.5 | 5 | 10 | 50 | 100 | 250 | 500 |
| MECPP | 0 | 2.5 | 5 | 10 | 50 | 100 | 250 | 500 |
| MEHHP | 0 | 2.5 | 5 | 10 | 50 | 100 | 250 | 500 |

| | | | | | | | | |
|---|---|---|---|---|---|---|---|---|
| MEOHP | 0 | 2.5 | 5 | 10 | 50 | 100 | 250 | 500 |

## Instrument conditions

Chromatographic conditions: the inlet temperature was 250°C, the shunt mode was no shunt, 99.999% helium was used as carrier gas and the flow rate was maintained at 1.2 ml/min. Autosampling was performed with a sample volume of 1 μL each time. 3 min, then the temperature was uniformly increased to 210 °C at the same rate, and finally to 280 °C at the same rate for 5 min, and the whole process took 33 min. Mass spectrometry (MS) conditions: The electron bombardment (EI) source was selected as the ion source for MS. The inlet temperature was 250 °C, the ion source temperature was 280 °C and the gain was 1. Characterisation: individual standards were processed into the sample and the retention time of each substance was characterised according to the product ion pair of each substance. See Table S6.

**Table S6.** Information sheet for on-board checking of metabolites and internal markers

| Metabolites and internal standard | retention time （RT） | parent ion (e.g. gas) | daughter ion (math.) | collision energy (physics)（CE） |
|---|---|---|---|---|
| 1-OHNAP-D7 | 15.987 | 223 | 208 | 15 |
| 1-OHNAP | 16.043 | 216 | 201 | 15 |
| 2-OHNAP | 16.547 | 216 | 201 | 15 |
| MMP | 16.731 | 237 | 89 | 15 |
| MEP | 17.837 | 251 | 75 | 15 |
| MIBP | 17.840 | 223 | 75 | 15 |
| MBP | 19.785 | 223 | 75 | 15 |
| 9-OHFLE | 20.231 | 254 | 165 | 20 |
| MOP | 20.426 | 223 | 73 | 15 |
| MEHP | 21.669 | 221 | 73 | 15 |
| 2-OHFLE | 22.471 | 254 | 239 | 15 |
| MEOHP | 24.633 | 221 | 73 | 15 |
| 4-OHPHE | 25.209 | 266 | 235 | 25 |
| 9-OHPHE | 25.211 | 266 | 73 | 25 |
| 1-OHPHE | 25.216 | 266 | 73 | 25 |
| 3-OHPHE | 25.854 | 266 | 73 | 25 |
| 2-OHPHE | 26.702 | 266 | 73 | 25 |
| MBZP | 27.261 | 179 | 105 | 20 |
| MEHP-C4 | 27.619 | 225 | 73 | 15 |
| MEHHP-C4 | 28.154 | 225 | 73 | 15 |
| MEHHP | 29.367 | 221 | 73 | 15 |
| 1-OHPYR | 30.857 | 290 | 73 | 25 |
| 1-OHPYR-D9 | 31.229 | 299 | 73 | 25 |

| | | | | |
|---|---|---|---|---|
| MECPP | 31.667 | 221 | 73 | 15 |
